# Supplementary material for: Brain Hsp90 Inhibition Mitigates Facial Allodynia in a Rat Model of CSD Headache and Upregulates Endocannabinoid Signaling in the PAG
Source: Pharmaceuticals (Basel). 2025 Sep 24;18(10):1430. doi: 10.3390/ph18101430 (PMC12567276; doi:10.3390/ph18101430)
Supplement: Supplementary file 1 [file pharmaceuticals-18-01430-s001.zip › pharmaceuticals-3844096-supplementary.pdf]

Figure	Groups	Number of animals
2B	vehicle	8
	1% DMSO	11
	17AAG (0.5 nmol)	12
	17AAG (5 nmol)	9
2C	V/aCSF	13
	17AAG (0.05)+aCSF	7
	17AAG (0.5)+aCSF	9
	17AAG (5)+aCSF	8
2D	V/KCl	12
	17AAG(0.5)+KCl	10
2F	V/KCl	11
	17AAG/KCl	11
	17AAG/KCl/Rimonabant	8
